# Plant community composition determines the strength of top-down control in a soil food web motif

**DOI:** 10.1038/srep09134

**Published:** 2015-03-16

**Authors:** Madhav Prakash Thakur, Nico Eisenhauer

**Affiliations:** 1 German Centre for Integrative Biodiversity Research (iDiv) Halle-Jena-Leipzig, Deutscher Platz 5e, 04103 Leipzig, Germany; 2 Institute of Biology, University of Leipzig, Johannisallee 21, 04103 Leipzig, Germany

## Abstract

Top-down control of prey by predators are magnified in productive ecosystems due to higher sustenance of prey communities. In soil micro-arthropod food webs, plant communities regulate the availability of basal resources like soil microbial biomass. Mixed plant communities are often associated with higher microbial biomass than monocultures. Therefore, top-down control is expected to be higher in soil food webs of mixed plant communities. Moreover, higher predator densities can increase the suppression of prey, which can induce interactive effects between predator densities and plant community composition on prey populations. Here, we tested the effects of predator density (predatory mites) on prey populations (Collembola) in monoculture and mixed plant communities. We hypothesized that top-down control would increase with predator density but only in the mixed plant community. Our results revealed two contrasting patterns of top-down control: stronger top-down control of prey communities in the mixed plant community, but weaker top-down control in plant monocultures in high predator density treatments. As expected, higher microbial community biomass in the mixed plant community sustained sufficiently high prey populations to support high predator density. Our results highlight the roles of plant community composition and predator densities in regulating top-down control of prey in soil food webs.

Trophic interactions in food webs are observed as a combination of top-down and bottom-up control among consumers and their resources that principally regulate population dynamics in ecosystems^1^,^2^. In tri-trophic interactions, either stronger top-down control (predator-induced control) or stronger bottom-up control (plant-induced control) of herbivore/detritivore populations have commonly been observed^3^,^4^. Moreover, recent studies have revealed that variations in plant community composition with respective changes in the basal resource regulate top-down control in ecological communities^5^,^6^.

Plant communities with a high number of species produce higher plant biomass providing sufficient energy to support greater communities of herbivores and detritivores, which cascade to higher densities of predators^6^,^7^, with a subsequent increase in top-down effects^7^. These findings of a productivity-driven proportional increase in the strength of trophic interactions at higher trophic levels agree with the classical Oksanen et al. (1981)^8^ hypothesis that predicts increased top-down control of prey by predators at high productivity (higher resource availability for prey). However, it is unclear how the relative importance of top-down and bottom-up control may change in ecosystems, which are experiencing shifts in plant community composition^9^ as well as declines in densities of predators^10^. Taken together, these two factors could potentially interact in complex ways and change the relative importance of top-down and bottom-up forces in influencing ecosystem processes. Here, we chose an experimental approach manipulating top-down forces in systems differing in productivity and illustrate that top-down control in a soil food web motif are contingent upon plant community composition.

In soil food webs, bottom-up forces through litter quantity and quality and microbial community biomass together with soil physio-chemical characteristics influence the strength of top-down control^2^,^11^. In the micro-arthropod sub-web of soil food webs, the main food source of prey communities like Collembola is soil microbial biomass^12^. At higher microbial biomass, many Collembola species are expected to thrive, which may simultaneously increase the density of their predators, such as predatory mites^13^. With an increase in prey density, both per capita and net prey suppression by predators get saturated^14^. However, saturation of predation also depends on the predator density, which co-determines prey suppression in combination with prey density^15^,^16^. For instance, at high predator density, antagonistic predator-predator interactions, such as interference and cannibalism, may reduce the ability of predators to suppress prey^17^. However, the magnitude of predator interference may decrease when the prey population is high and heterogeneously distributed^18^.

Plant communities fuel soil food webs via litter and dead root inputs, together with rhizodeposits that plant roots release into soil during plant growth^19^,^20^. These organic inputs collectively represent the key resource for microbial community biomass in soil. However, several studies have shown variations among plant community compositions and among plant species in fueling soil microbial biomass and related food webs^21^,^22^. Plant mixtures with functionally different species were shown to enhance microbial biomass in soil^23^,^24^. Furthermore, certain plant functional groups are associated with higher microbial biomass due to their positive association with N-fixing bacterial species (legumes)^25^ or due to high root biomass (grasses)^26^. Functionally different plant groups also create heterogeneous environments in soil^27^ that may favour predators in suppressing prey^5^,^28^.

In this research, we expect predator density (top-down force) and plant community-induced variations in soil microbial biomass (bottom-up force) to interactively affect prey populations. Higher densities of predators can increase the suppression of prey; however, only when the prey has sufficient amounts of resources to exploit, which would depend on plant community composition. Therefore, we hypothesize that the strength of top-down control will increase with predator density in the mixed plant community, but will decline with predator density in monoculture plant communities (Figure 1)^5^. We test our hypotheses in a soil micro-arthropod predator-prey system with Collembola as prey species and predatory mites as predators. This soil food web motif was studied in different plant communities, consisting of a mixed plant community with three plant functional groups (grass, herb, and legume) together and the respective monocultures.

## Results

**Figure 2:**
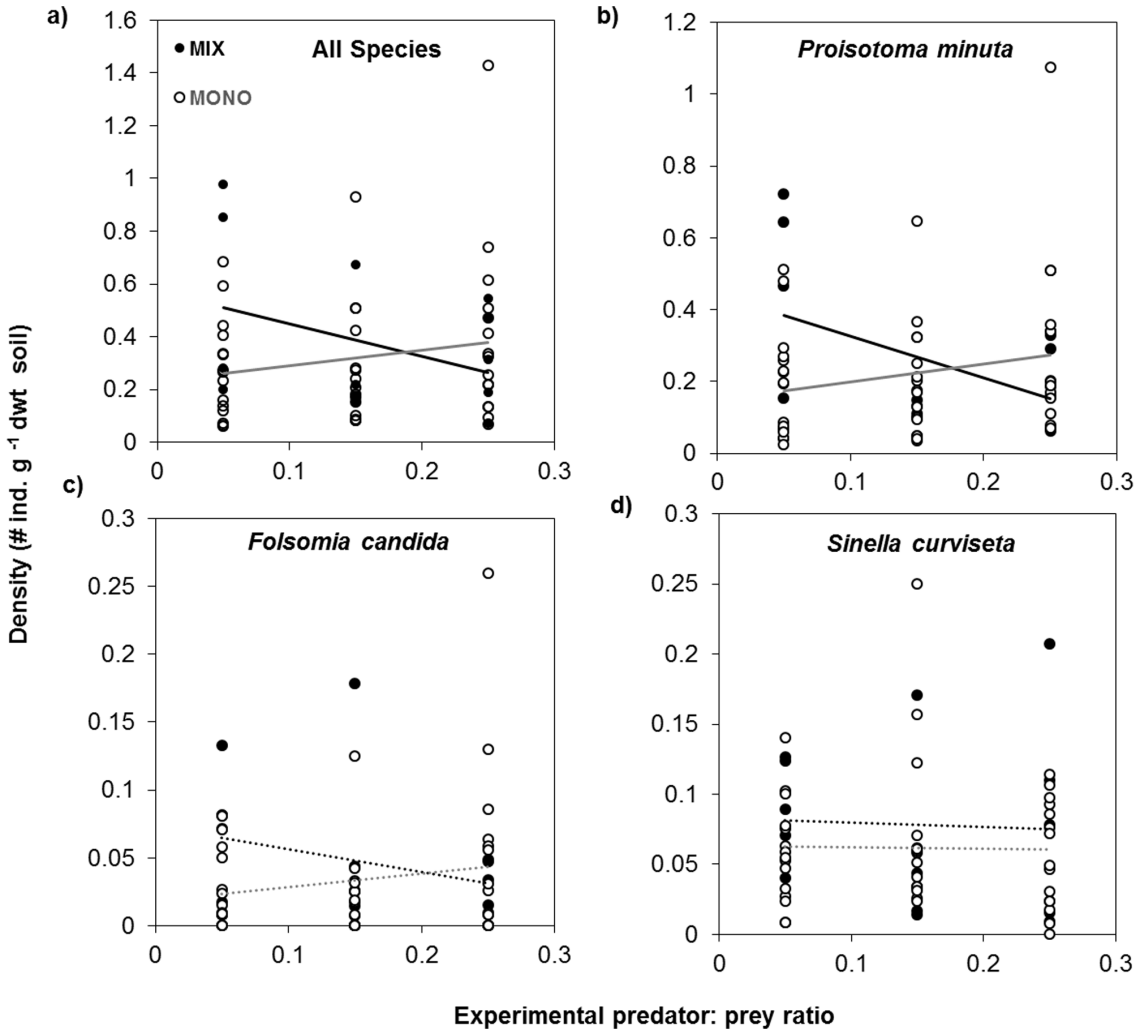
(a) Total Collembola densities, (b) densities of *Proisotoma minuta*, (c) densities of *Folsomia candida*, and (d) densities of *Sinella curviseta* at the end of the experiment as affected by experimental predator densities (indicated by predator: prey ratio) and plant community composition. The black solid lines show significant relationships, whereas dotted lines indicate non-significant relationships.

We found no significant main effects of predator density (GLMM, F = 0.09, P = 0.55) and plant community composition (GLMM, F = 1.31, P = 0.63) on Collembola density. However, and in line with our expectation, we found a significant interaction effect of predator density and plant community composition on total Collembola density (GLMM, F = 4.18, P = 0.02). In treatments with high predator densities, total Collembola densities decreased in the mixed plant community, while they increased in monoculture plant communities (Figure 2a). The interactive effect of predator density and plant community composition on total Collembola density was driven by the response of *Proisotoma* *minuta -* the smallest body-sized prey species in our experiment (GLMM, F = 5.62, P = 0.01; Figure 2b). *Folsomia candida* showed a similar response, but the interaction effect was not significant (GLMM, F = 2.40, P = 0.12; Figure 2c). By contrast, *S. curviseta* showed no response to the interaction between predator density and plant community composition (GLMM, F = 0.008 and P = 0.9; Figure 2d). Further, we also found no interactive effects of predator density and plant monocultures on Collembola species (*Proisotoma*: F = 0.10, P = 0.90; *Folsomia*: F = 0.32, P = 0.72; *Sinella*: F = 0.16, P = 0.84) (Supplementary Figure S1).

**Figure 3:**
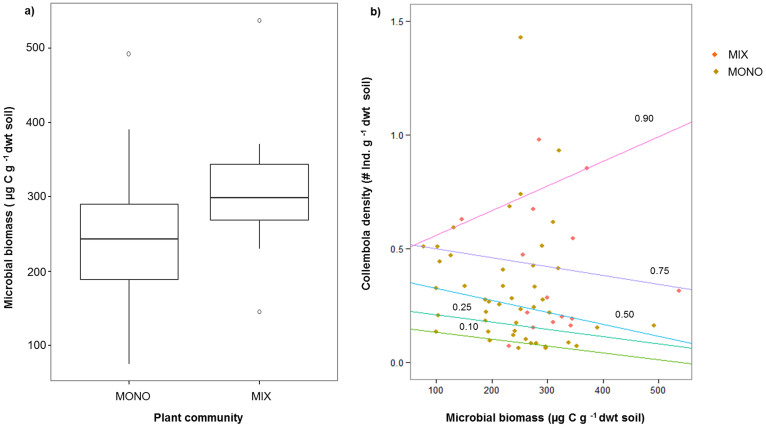
(a) Soil microbial biomass C as affected by plant community composition. (b) Relationships between total Collembola densities and soil microbial biomass C at different quantiles.

In line with our expectations, soil microbial biomass C was significantly higher in the mixed plant community compared to monoculture plant communities (+30%; LMM, F = 8.13, P <0.01; Figure 3a), but did not vary significantly among the different monocultures (LMM, F = 2.12, p = 0.11, Supplementary Figure S2). Predator density had no significant effect on soil microbial biomass C (LMM, F = 0.05, P = 0.82). In addition, we found a marginally significant interactive effect of plant community composition and predator density on soil microbial biomass C (LMM, F = 2.92, P = 0.08). Microbial biomass C increased in the mixed plant community compared to the soil at the start of the experiment (+7%), whereas it decreased in monoculture plant communities (−17%).

Results from quantile regression showed that at the higher quantile (0.90) of total Collembola density, soil microbial biomass and Collembola density were positively associated (Figure 3b). On the contrary, at lower quantiles, soil microbial biomass and Collembola density showed a negative relation (Figure 3b). The non-parametric quantile regression tests revealed significant associations between soil microbial biomass and Collembola density at quantiles 0.90 (P = 0.04), 0.75 (P <0.01), and 0.50 (P <0.001), whereas associations were not significant at quantiles 0.25 (P = 0.13) and 0.10 (P = 0.52).

## Discussion

Our results show two contrasting patterns of top-down control of prey populations based on experimentally varied predator density and plant community composition. Top-down control of Collembola increased (i.e., decrease in Collembola density) at high predator density in the mixed plant community, but not in monoculture plant communities, which agrees with our hypothesis. We speculate that the contrasting predator density effects on suppression of Collembola populations were partly due to effects mediated by plant community on soil microbial community biomass.

Microbial biomass was significantly higher in the mixed plant community than in monoculture plant communities (Figure 3a). Moreover, the positive association of higher Collembola population density and higher microbial community biomass indicates that the higher availability of microbial biomass supported higher Collembola population densities (Figure 3b). Although correlative, this relation provides some evidence that the higher sustenance of the prey community was possible only when the basal resource level was high. Hence, due to favourable conditions (higher microbial biomass) for Collembola population in the soil of the mixed plant community, only a higher predator density was able to exert top-down control, but not the low predator density treatment.

Our results support the hypothesis of increased trophic control of prey by predators as productivity increases^8^,^29^. Furthermore, our results show interactive effects of predator density and productivity co-determining changes in prey populations. This is also in accordance to studies showing interactive effects of top-down and bottom-up forces in regulating food web structure^30^,^31^. Although, most of these previous studies demonstrated the influence of a top-down force by manipulating the presence/absence of predators, our results show that differences in predator density can alter food web structure depending on the availability of the basal resources.

In contrast to the results in the mixed plant community, interference among predatory mites at high predator density may have increased in monoculture plant communities due to low Collembola density causing a weak top-down control (Figure 2a). Interference among predatory mite individuals are argued to be low at sufficiently abundant prey populations due to their ability of dispersing into prey clusters^18^. This could cause a higher suppression of prey, as in the case of high density predator treatments in the mixed plant community in this study.

Mixed plant communities in combination with different functional groups including legumes and grasses have been shown to increase Collembola densities, likely due to elevated microbial biomass in soil^32^,^33^. Due to higher production of fine roots and an associated increase in rhizodeposition, mixed plant communities potentially provide favourable microhabitat conditions in soil for microbial growth^26^,^34^.

To overcome predation pressure, prey communities may show compensatory population growth via faster regeneration^35^. In order to do so, Collembola species are expected to increase their grazing activity on microbial communities^36^. Increased Collembola grazing potentially can decrease microbial biomass; however, our finding suggest that this was not the case, at least in the mixed plant community. It is important to note though that our study design did not allow us to test how Collembola grazing pressure could have affected microbial biomass (Supplementary Figure S3). Some studies have reported that Collembola grazing could essentially influence soil microbial biomass^36^,^37^; however, less is known about how such grazing effects could occur in the context of different plant communities^38^. Our results also show that realized predator density was higher in the mixed plant community independent of predator density treatments at the final harvest supporting the notion that plant species mixtures support higher predator densities than monocultures^5^,^6^ (Supplementary Figure S4).

The significant suppression of only *P. minuta* by predators among the three studied Collembola species indicates a combination of strong and weak trophic interactions in our study depending on Collembola species identity (Figure 2b). A plausible reason for strong suppression of *P. minuta* could be their relatively small body size, which may cause higher foraging advantages, such as reduced handling time, for predators compared to the large-sized prey such as *S. curviseta*^39^. Interestingly, when changes in Collembola density from the start of the experiment to the final harvest were compared, *P. minuta* density also increased more than the other two Collembola species (see differences in Y-axis scales in Figure 2b, c, and d). This further indicates compensatory population growth in *P. minuta*, which could occur in prey communities when exposed to high predation pressure^35^,^40^. Species undergoing faster regeneration like *P. minuta*^41^ often show compensatory patterns and are therefore superior at exploiting available resources^42^. At high predator density, an increase in prey density would lead to a higher probability of predator-prey encounters, which could have contributed to the observed suppression of *P. minuta* in the resource-rich environment of the mixed plant community.

Spatial and resource heterogeneity in soil have been shown to be higher in diverse plant communities than in monocultures^43^. We observed consistent patterns of soil microbial biomass and prey population among monocultures of the three plant functional groups at different predator density treatments (Supplementary Figure S1), which is inconsistent with studies that have reported stronger plant identity effects on the trophic structure of soil food webs due to plant species-specific soil environments^44^. The plant species that we used have different root architectures and biomass^45^, and variations in plant-derived organic inputs in the mixed plant community^25^ may have created a more heterogeneous environment^46^ in soil promoting prey suppression by predators. Such heterogeneous environments will enhance the clustering of Collembola species in resource-rich patches (higher microbial biomass) that in general would favour predation by predatory mites^18^. However, as we were unable to show such direct links in the present study, the proposed relationship between resource heterogeneity and predator-prey interactions in soil merits further exploration.

In general, at high predator density, prey suppression can increase for a short duration when prey population growth is constrained by a lack of the basal resource^47^. It is important to note that when resource availability limits prey population growth, predator density would decline due to starvation-induced mortality. This could be one reason for weak effects of initial predator densities on the realized predator densities compared to differences between the mixed and monoculture plant communities (Supplementary Figure S4). In low productive systems (monoculture plant communities), high predator density might have suppressed Collembola population initially, but as the experiment progressed, prey suppression may have decreased as Collembola population did not thrive as much as in the mixed plant community, consequently leading to the decline in predator densities. Multiple harvests or developing procedures to temporally track population dynamics^48^ in such experiments can provide further insights into density effects of predators on prey suppression as influenced by resource availability.

Prey suppression by predators in ecosystems contribute to many ecosystem functions, such as nutrient cycling^49^. Predators are the most vulnerable trophic group to the effects of disturbances, such as land fragmentation and climate warming^10^,^50^. A decline in predator density can detrimentally affect ecosystem processes through trophic cascades^49^. Utilizing a soil food web motif, our study shows that the top-down control of prey depends on the availability of the basal resource as well as on the density of predators. We argue that such interactive effects of predator density (top-down force) and productivity or resource availability mediated via the plant community (bottom-up force) play a crucial role in structuring food webs. Since both top-down and bottom-up factors determining food web structure are subjected to acute and chronic anthropogenic perturbations, our study highlights that such interactions are crucial to understand and predict changes in food web structure and concomitant ecosystem functions.

## Methods

### Microcosm set-up

The experiment was conducted in microcosms made of polyvinyl chloride (PVC) tubes (height: 10 cm and diameter: 7 cm) with the soils (pH = 8.1, C: N ratio = 15.7) from a field site adjacent to the Jena Experiment (floodplain grassland)^51^. The soil was sieved using a 2 mm-mesh and defaunated by two cycles of the thaw-rethaw method in which soils were initially frozen at −20°C for 48 hours and later defrozen at room temperature for several days^52^. This method has been shown to remove micro-arthropods and even nematodes from soil without affecting microbial communities^52^. We added 300 g of this defaunated soil to microcosms together with 500 mg of grass root litter (*Lolium perenne*) and incubated that for two weeks with 10 ml tap water added every day in order to facilitate microbial colonization. The same amount of water was added every day throughout the experiment.

### Plant communities

Mixed and monoculture plant communities were established in the defaunated soils, which had been incubated with grass litter for 2 weeks. The mixed plant community included *Trifolium pratense* (legume), *Poa pratensis* (grass), and *Rumex acetosa* (herb), and monoculture plant communities consisted of those three plant species alone in the microcosms. These plant species have been shown to vary in their root traits such as root depth and are part of plant species pool of the Jena Experiment^45^,^51^. Seeds of these plant species (obtained from Rieger-Hoffmann GmbH, Blaufelden-Raboldshausen, Germany) were sown separately in the same defaunated soil and transplanted into the microcosms after six weeks of germination (seedlings were chosen with similar heights of about 5–8 cm). Three plant individuals from one species were planted into monoculture microcosms (establishing three different monoculture treatments), whereas one individual per plant species was planted to construct a mixed plant community. To give plant communities sufficient time to establish in the microcosms, Collembola were added only after 20 days from the plant transplantations into the microcosms.

### Soil microarthropod community

The prey community consisted of three Collembola species (*Proisotoma minuta, Folsomia candida*, and *Sinella curviseta*), which were added to the microcosms in equal densities (details below). Families of these species (*P. minuta*: Isotomidae, *F. candida*, and *S. curviseta*: Entomobryidae) are reported in the soil of the Jena Experiment^33^, while the used species are easy to culture in labs. All three species were cultured at the University of Jena at 20°C by feeding with dry yeast. These three species vary in their body size, with *P. minuta* (mean body size: 1.1 mm)^53^ being the smallest, *F. candida* (mean body size: 1.59 mm)^54^ being of intermediate size, and *S. curviseta* being the largest species (mean body size: 2 mm)^54^. Collembola species feed on several substrates such as fungi, bacteria and litter, but they are commonly accepted as microbial feeders^53^,^55^. This main feeding behavior of the three species was confirmed as they were cultured with dry yeast. A generalist predatory mite (*Hypoaspis aculeifer*, mean body size: 0.6 mm)^56^ was used as the model predator (purchased from Schneckenprofi in Germany). *Hypoaspis aculeifer* are common soil dwelling predators and are common predators of Collembola^57^.

Each microcosm received 20 individuals of each Collembola species (in total 60 individuals of Collembola, i.e., 0.20 ind. per g dry weight of soil), and one of three different densities of predators: 3, 9, and 15 individuals (i.e., 0.01, 0.03, and 0.05 ind. per g dry weight of soil). The Collembola density used in this experiment (approx. 20,000 ind/m^2^) is comparable to the field density in the Jena Experiment^58^. Predators were added 6 days after the addition of Collembola. We had four plant combinations (one mixed plant community and three different monocultures) crossed with three predator density treatments, each replicated 5 times (60 microcosms in total). The microcosms were randomly arranged in a block design to account for variations in wind flow from the cooling fan used to regulate temperature in the greenhouse. We applied a day/night cycle of 16 (20°C)/8 hours (16°C).

The experiment ran for 57 days after the day of predator addition. The experimental duration was adequate for regeneration of Collembola species^59^ and the predatory mite^60^. During final harvest, soil cores (5 cm deep, 5 cm in diameter) were taken from microcosms to extract Collembola and predatory mites using heat extraction^61^. During this extraction, soil cores were gradually heated for 10 days from 25°C up to 50°C. Collembola and predatory mites were collected in 70% ethanol and subsequently counted under a dissecting microscope.

### Microbial biomass

We took 5 g of sieved soils (2 mm mesh size) from microcosms to estimate microbial biomass carbon (C) using an O_2_-microcompensation apparatus^62^. This method does not provide information on the composition of the soil microbial community, but captures the active part of the microbial community very efficiently^62^, which we used as a proxy of resource availability for Collembola in soil. Besides, plant-derived carbon inputs in soil are often positively correlated to microbial biomass C^63^. Microbial biomass was determined at the start of the experiment after 2 weeks of soil incubation with litters (before plants were transplanted into the soil) and at the final harvest. Microbial respiration was measured at hourly intervals for 24 hours at 19°C. Afterwards, substrate-induced respiration was measured by adding D-glucose as a substrate for about 10 h at 19°C. The mean of the three lowest readings within the first 10 hours was assessed as the maximum initial respiratory response (MIRR), and microbial biomass (μg C g^−1^ soil dry weight) was calculated by multiplying MIRR with correction factor of 38^64^.

### Statistical analysis

Generalized linear mixed models (GLMM) were used to test the treatment effects of predator density (as experimental predator: prey ratio; linear term with three levels) and plant community composition (as mixed plant community and monoculture; categorical term with two levels) on Collembola density with blocks as the random effect. GLMM are generally recommended for regressions with the count data^65^. Further, due to over-dispersion in the Collembola count data (i.e., residual deviance = 1248.1≫ degrees of freedom = 55), we tested quasi-poisson vs. negative binomial distributions model fits for variations in Collembola density and selected the model with the lower value of maximum likelihood estimates^66^. The model with negative binomial errors (maximum likelihood estimate = 270.83) fitted better than the model with quasi-poisson errors (maximum likelihood estimate = 680.47). Although, model comparison was carried out with absolute counts of Collembola, we report GLMM (negative binomial error) results for Collembola counts per dry weight of soil (used during the animal extraction) due to further improved model fit (AIC_absolute counts_ = 553.67> AIC_per g dry weight of soil_ = 86.08). For species-specific responses of Collembola to plant community compositions (mixed vs. monoculture) and predator density, we again used GLMM with negative binomial errors. Further, we tested if soil microbial biomass C was influenced by plant community composition and predator density treatments. For this, we used linear mixed effect models (LMM) with blocks as the random effect. We also tested whether plant monocultures and predator densities affected Collembola density (GLMM for count data, negative binomial error) and microbial biomass C (LMM for biomass data). We correlated microbial biomass C with Collembola density using quantile regression due to over-dispersion of Collembola density data^67^. We used quantile regression at quantiles 0.10, 0.25, 0.50, 0.75, and 0.90. Quantile regression incorporates the unequal variances of the response variables and is useful to consider the possibility of multiple slopes for minimum to maximum responses^67^. All statistical analyses were carried out in R statistical software version 2.15.2 (R Development Core Team, 2012).

## Supplementary Material

Supplementary Information Supplementary information

## Figures and Tables

**Figure 1:**
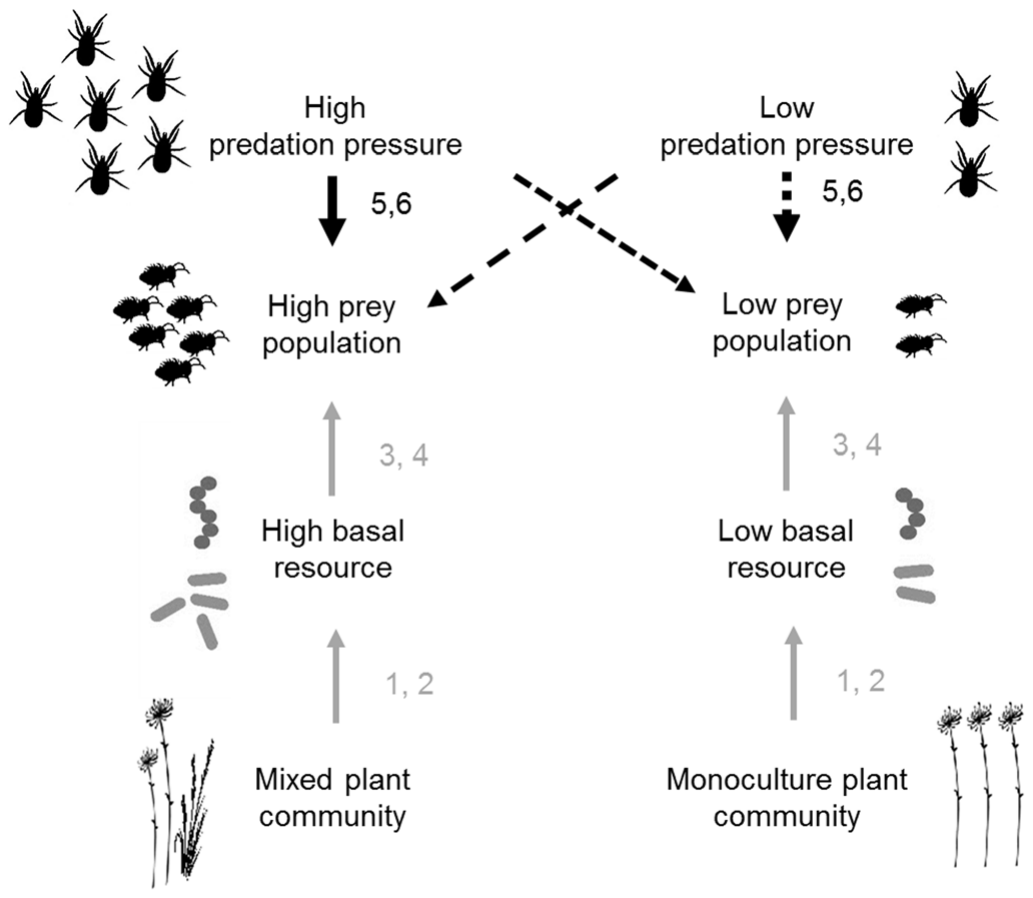
Conceptual diagram illustrating the working hypotheses. The basal resource in this study is soil microbial biomass C. The upward directing gray arrows with numbers indicate literature-based information for the shown relation. For instance, mixed plant communities have been shown to be associated with higher soil microbial biomass C (Reference 1). The downward directing arrows indicate hypotheses on differences in the strength of top-down control at high vs. low predator densities in two plant community composition scenarios (mixed and monocultures). The dashed arrows indicate weak top-down control, whereas solid arrows indicate strong top-down control. The crossed dashed arrows indicate weak top-down control as predators at high density will not thrive in the presence of low prey density, and low predator densities cannot suppress high densities of prey (see text for details). Figure is drawn by MPT. References: 1 - Spehn et al. 2000^23^; 2 - Eisenhauer et al. 2013^24^; 3 - Bonkowski et al. 2000^12^; 4 - Scheu et al. 2005^13^; 5 - Oksanen et al. 1981^8^; 6 - Haddad et al. 2011^7^.
