# Supplementary material for: Plant community composition determines the strength of top-down control in a soil food web motif
Source: Sci Rep. 2015 Mar 16;5:9134. doi: 10.1038/srep09134 (PMC5390908; doi:10.1038/srep09134)
Supplement: Supplementary Information [file srep09134-s1.pdf]

Supplementary Figures

Plant community composition determines the strength of top down control in a soil food web motif

Madhav Prakash Thakur and Nico Eisenhauer

.

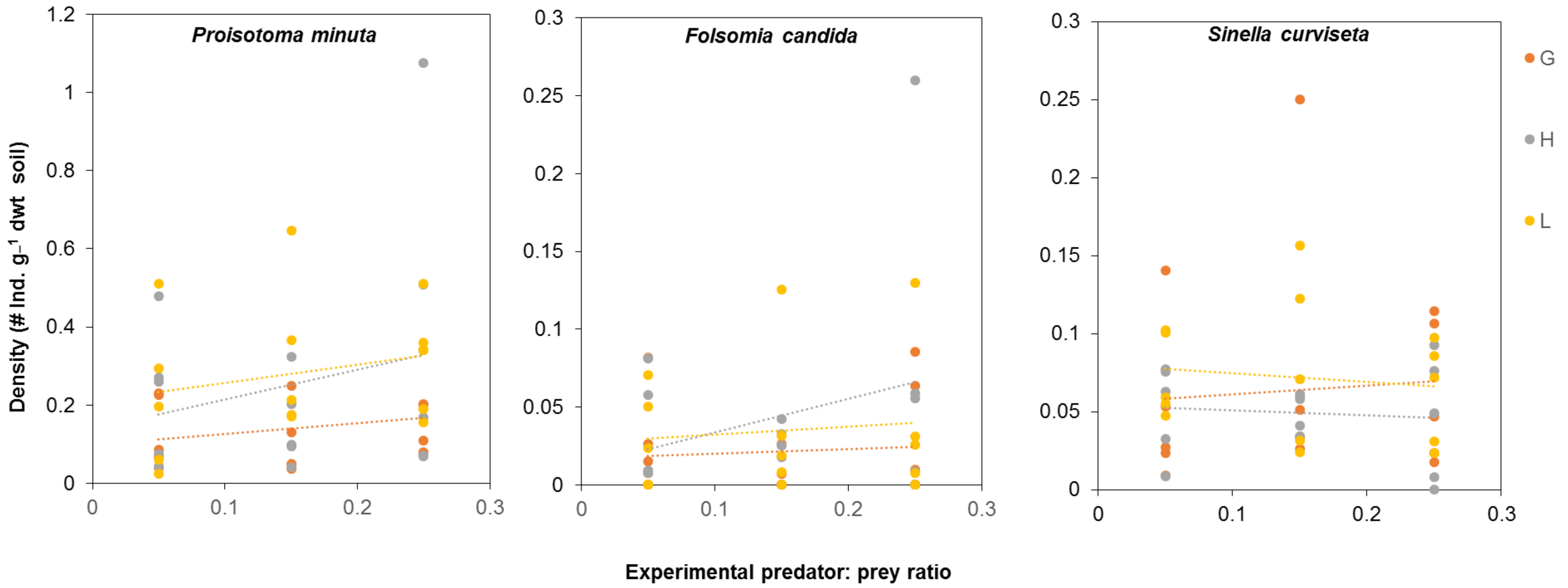

Figure S1: Species-specific responses of Collembola to increasing predator densities in different plant monocultures (H: herb monoculture, G: grass monoculture, and L: legume monoculture). Using GLMM (negative binomial fits), we found no significant interaction between plant monoculture communities and predator density for all three Collembola species (*Proisotoma*: F=0.10, P=0.90; *Folsomia*: F=0.32, P=0.72; *Sinella*: F=0.16, P=0.84). Only *Proisotoma* densities were marginally significantly different among plant monocultures (F=2.82; P=0.06)

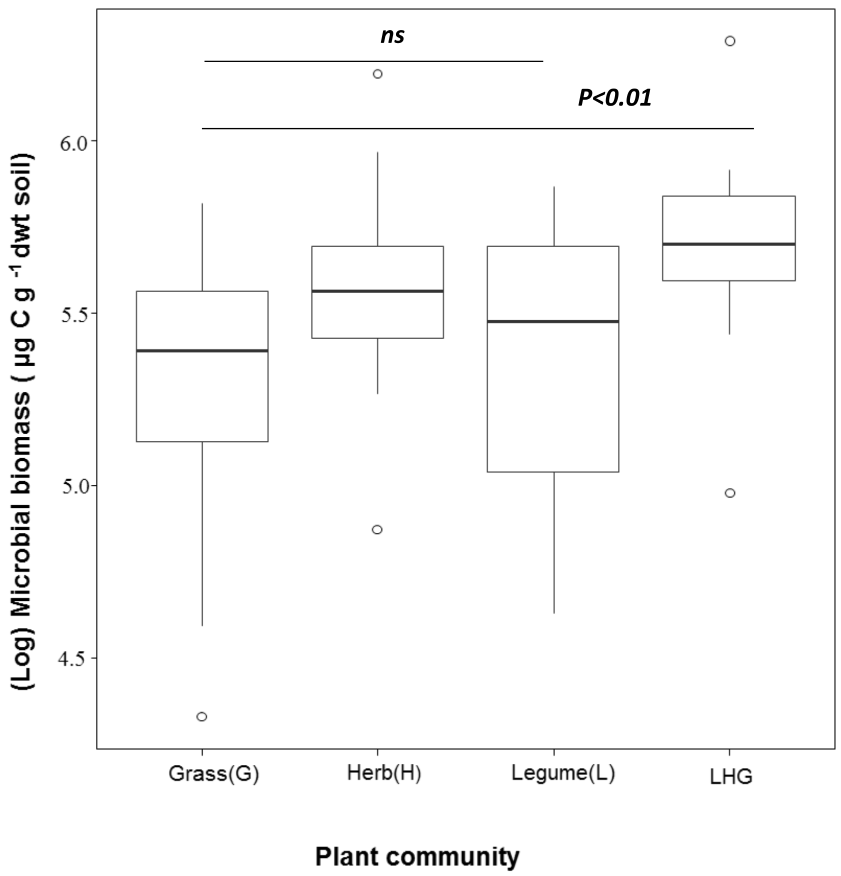

Figure S2: Microbial biomass C across three plant monocultures and a mixture community. Plant monocultures did not significantly differ indicated by "ns" in the figure above whereas plant mixture and plant monocultures significantly differed with higher microbial biomass C in mixture plant community (LHG).

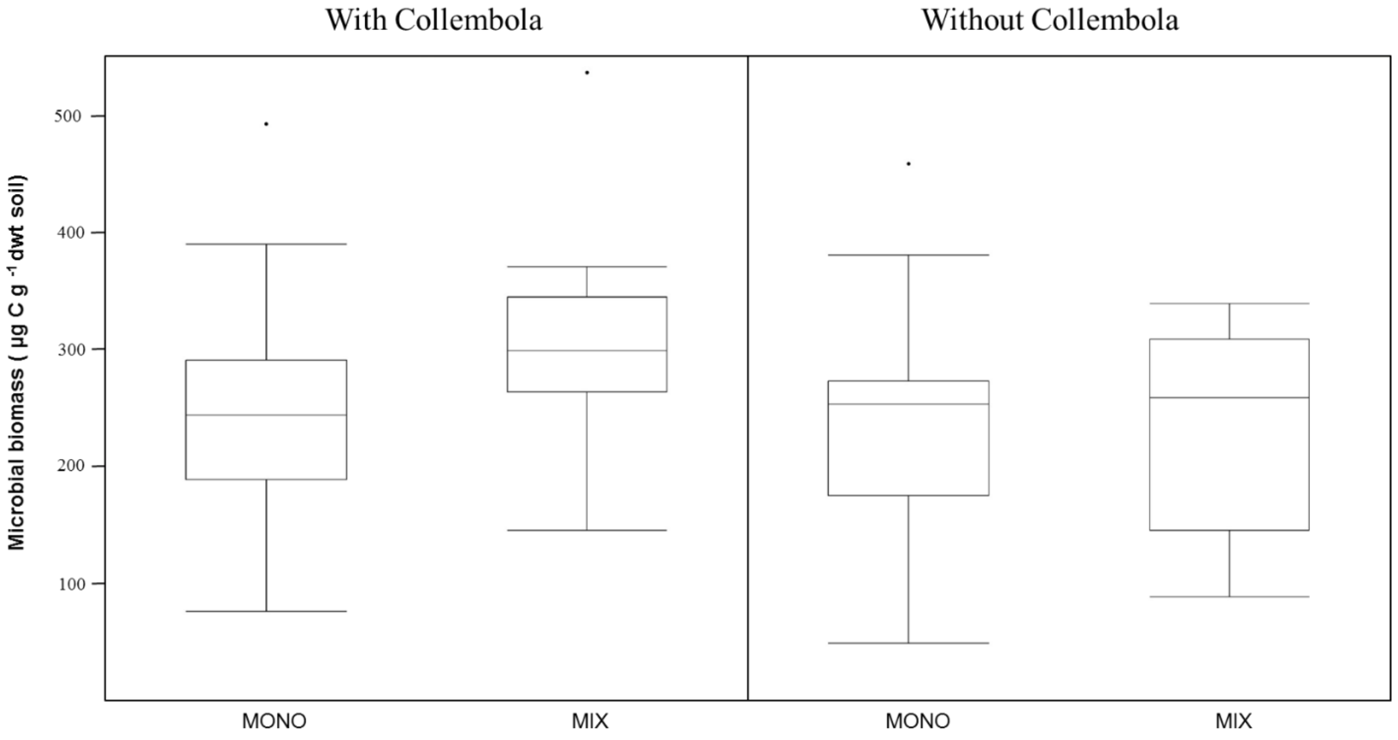

Figure S3: Microbial biomass with and without Collembola in monoculture and mixed plant community. We ran a two-way ANOVA model to analyze whether presence and absence of Collembola in monoculture and mixed plant community had any effect on microbial biomass (log-transformed in the analysis). Our results show no significant effect of Collembola (p-value=0.22, t=1.23), plant community (p-value=0.26, t=1.12) and no interaction effects between Collembola presence and plant community composition (p-value=0.14, t=1.47). Please note that we do not have an absolute control of Collembola effect, i.e. “with Collembola” treatment in the figure above are crossed with predator density. Hence, an absolute effect of Collembola on microbial biomass could not be tested from our experimental design.

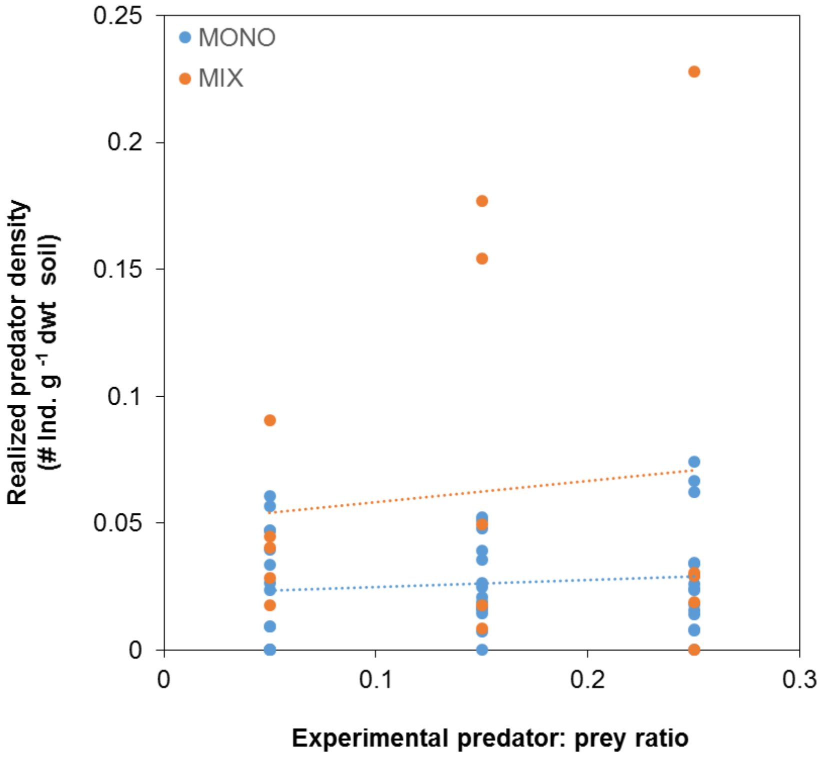

Figure S4: Predator density at the final harvest. We only found significant plant community effects on the realized predator density (F=4.56, P=0.003) using GLMM (negative binomial fit). Initial experimental predator density (F=0.73, P>0.05) and the interaction between plant community and initial experimental predator density (F=0.64, P>0.05) both had non-significant effects on the final predator density. Mixture plant communities had 58% higher absolute predator densities than in monoculture plant communities when averaged over all experimental predator density treatments
